# Bone marrow concentrate-induced mesenchymal stem cell conditioned medium facilitates wound healing and prevents hypertrophic scar formation in a rabbit ear model

**DOI:** 10.1186/s13287-019-1383-x

**Published:** 2019-08-28

**Authors:** Ching-Hsuan Hu, Yi-Wen Tseng, Chih-Yung Chiou, Kuan-Chun Lan, Chih-Hung Chou, Chun-San Tai, Hsien-Da Huang, Chiung-Wen Hu, Ko-Hsun Liao, Shiow-Shuh Chuang, Jui-Yung Yang, Oscar K. Lee

**Affiliations:** 1 Department of Plastic and Reconstructive Surgery, Chang Gung Memorial Hospital, Chang Gung Medical College and Chang Gung University, Taoyuan, Taiwan; 2 Institute of Clinical Medicine, National Yang-Ming University, Taipei, Taiwan; 3 Stem Cell Research Center, National Yang-Ming University, Taipei, Taiwan; 4 Institute of Molecular Medicine and Bioengineering, National Chiao Tung University, Hsinchu, 300 Taiwan; 5 Department of Biological Science and Technology, Center for Intelligent Drug Systems and Smart Bio-devices (IDS²B), National Chiao Tung University, Hsinchu, 300 Taiwan; 6 Department of Biological Science and Technology, National Chiao Tung University, Hsinchu, 300 Taiwan; 7 Warshel Institute for Computational Biology, School of Life and Health Sciences, School of Sciences and Engineering, The Chinese University of Hong Kong, Shenzhen, 518172 China; 8 Department of Public Health, Chung Shan Medical University, Taichung, 402 Taiwan

**Keywords:** Hypertrophic scar, Bone marrow concentrate, Mesenchymal stem cell-conditioned medium

## Abstract

**Background:**

Hypertrophic scars (HSs) are formed via an aberrant response to the wound healing process. HSs can be cosmetic or can result in functional problems. Prolonged proliferation and remodeling phases disrupt wound healing, leading to excessive collagen production and HS formation. However, there are currently no satisfactory drugs to prevent HS formation. Mesenchymal stem cell (MSC) conditioned medium (CM) has therapeutic effects on wound healing and preventing HS formation. Bone marrow concentrate (BMC) contains various growth factors and cytokines that are crucial for regeneration and has been applied in the clinical setting. In this study, we evaluated the effects of BMC-induced MSC CM on HS formation in a rabbit ear model.

**Methods:**

We established a rabbit ear wound model by generating full-thickness wounds in the ears of rabbits (*n* = 12) and treated wounds with MSC CM, BMC CM, or BMC-induced MSC CM. Dermal fibroblasts from human hypertrophic scar were stimulated with transforming growth factor beta 1 (TGF-β1) for 24 h and cultured in each culture medium for 72 h. We measured the hypertrophic scar (HS) formation during the skin regeneration by measuring the expression of several remodeling molecules and the effect of these conditioned media on active human HS fibroblasts.

**Results:**

Our results showed that BMC-induced MSC CM had greater antifibrotic effects than MSC CM and BMC CM significantly attenuated HS formation in rabbits. BMC-induced MSC CM accelerated wound re-epithelization by increasing cell proliferation. Additionally, BMC-induced MSC CM also inhibited fibrosis by decreasing profibrotic gene and protein expression, promoting extracellular matrix turnover, inhibiting fibroblast contraction, and reversing myofibroblast activation.

**Conclusions:**

BMC-induced MSC CM modulated the proliferation and remodeling phases of wound healing, representing a potential wound healing agent and approach for preventing HS formation.

**Electronic supplementary material:**

The online version of this article (10.1186/s13287-019-1383-x) contains supplementary material, which is available to authorized users.

## Introduction

Hypertrophic scars (HSs) are a common complication of burns and other soft tissue injuries. After injury to dermal tissues, HSs can occur owing to fibroblast proliferation, inflammatory cell infiltration, and abnormal extracellular matrix (ECM) accumulation and remodeling, particularly with regard to collagen [1]. Several treatments for HSs, including surgical excision, intralesional corticosteroid injection, compression, laser treatment, and interferon injection, have been developed to date [1]. However, these treatments are not effective for preventing excessive scar tissue formation and regenerating healthy tissue. Hence, management of HS formation remains a challenge.

Bone marrow concentrate (BMC) has been used extensively in regenerative medicine [2]. BMC is an autologous bone marrow-derived product that provides a heterogeneous mixture of cells and a variety of bioactive growth factors and cytokines associated with wound healing [3]. BMC is also easy to collect, enabling immediate treatment of injuries [3]. BMC has been applied in patients with ischemic wounds, osteoporosis, arthritis, and tendon healing, yielding promising outcomes [2–6]. Although the specific mechanisms through which BMC affects wound healing have not yet been well established, several mechanisms have been proposed, including increasing tissue vascularity and tissue microperfusion at the capillary level, remodeling of fibrotic tissues to allow new capillary growth or to increase interstitial fluid flow, and modulating the inflammatory response [5].

MSCs are multipotent with self-renewal and multiple differentiation capacities. MSCs are promising in a variety of antifibrosis applications owing to their effects on attenuating collagen deposition [7]. Paracrine mechanisms mediate the therapeutic effects of MSCs, and trophic factors secreted from MSCs suppress inflammation and apoptosis, but promote angiogenesis and mitosis in parenchymal cells [8]. MSCs and BMC are effective for accelerating wound healing. However, it is unclear whether BMC can promote the therapeutic effects of MSCs on wound healing and HS formation. The bioactive cytokines in BMC may enhance MSC regenerative capacity to prevent HS formation.

Accordingly, in this study, we aimed to validate the therapeutic effects of conditioned medium (CM) from BMC-induced MSCs on HSs in a rabbit model.

## Materials and methods

### Patients and tissue samples

All HS tissues were harvested at the time of surgery from four patients confirmed to have clinical and pathological evidence of HSs and who had not yet received scar treatment. Before surgery, all patients provided verbal and written consent to take part in the study. The study was approved by the Medical and Ethics Committee of our hospital (Institutional Review Board approval number: 201701193B0).

### Isolation and culture of scar-derived fibroblasts

Dermal fibroblast cultures were established from tissue specimens, which were processed within 4 h after surgical excision, as previously described [9]. Briefly, the epidermis of specimens was removed, and the dermis was minced into small pieces (1 mm^3^). The dermis was repeatedly washed in sterile Dulbecco’s modified Eagle’s medium (DMEM; Gibco, NY, USA) supplemented with 100 U/mL penicillin, 100 mg/mL streptomycin, and 10% fetal bovine serum (FBS; Gibco). The specimens were then exposed to 10 mg/mL collagenase type IV (Sigma-Aldrich, MO, USA) and 10 mg/mL Dispase II (Invitrogen, CA, USA) in DMEM and incubated at 37 °C in a 5% CO_2_ atmosphere for 3 h. After enzymatic digestion, the cell suspension was filtered through a 70-μm cell strainer (BD Biosciences, CA, USA) and centrifuged at 200×*g* for 4 min. The supernatant was discarded, and the cell pellet was resuspended in sterile DMEM (Gibco) supplemented with 100 U/mL penicillin, 100 mg/mL streptomycin, and 10% FBS (Gibco). The cells were cultured in 100-mm plastic tissue culture dishes (BD Biosciences). Once confluent scar-derived fibroblasts were established as monolayer cultures, cell passaging was performed using 0.125% trypsin (Gibco). Scar-derived fibroblasts between the sixth and 11th passages were used for experiments.

### Isolation of mouse bone marrow MSCs

The isolation of mouse MSCs from the bone marrow of Balb/c mice and characterization of MSCs were identified with our previously reported method [10–13]. Mouse MSCs were then cultured in low-glucose DMEM (Sigma-Aldrich) supplemented with 10% FBS (Thermo Fisher Scientific, Waltham, MA, USA) and 1% penicillin-streptomycin-glutamine (Thermo Fisher Scientific). The quality and purity of obtained MSCs were assessed, and the isolated MSCs were able to be differentiated into the bone, adipose tissue, and hepatocyte in vitro (see Additional file 1) [10–13]. MSCs were used at passages 8–13 in this study. The study was approved by the Medical and Ethics Committee of our hospital (approval no. 201701193B0).

### BMC preparation

Bone marrow aspiration, concentration, harvesting, and processing were performed as described previously, with modifications [4, 14]. Before aspiration, a 10-mL syringe was flushed with heparin (1000 μ/mL). After adequate anesthesia and sterile conditions, the iliac crests of rabbits were penetrated with an 18-gauge needle and aspirated. The aspirate was poured into a 10-mL centrifuge tube and spun at 1200×*g* for 10 min. After completion of this process, the buffy coat layer and platelet-poor plasma layer were extracted from the centrifuge tube and discarded. Secondary centrifugation was performed at 2400×*g* for 6 min. Clear supernatants were removed by aspiration until only 1 mL was left.

### Preparation of MSCs, BMC CM, and BMC-induced MSC CM

When mouse bone marrow MSCs reached approximately 80% confluence in 10-cm dishes, culture medium was changed to 10 mL DMEM. For the preparation of BMC-induced MSC CM, 200 μL BMC was added to cultures of MSCs. For the preparation of BMC CM, 200 μL BMC was added to 10-cm dishes containing 10 mL DMEM. All culture media were separated after 48 h and used as MSC CM, BMC-induced MSC CM, and BMC CM. This procedure was repeated three times; CM from three different time points was pooled and filtered through a 0.22-μm filter (Millex-GP syringe filter; Millipore, Billerica, MA, USA). Pure DMEM was used as a control medium.

### Rabbit ear HS model

**Fig. 1:**
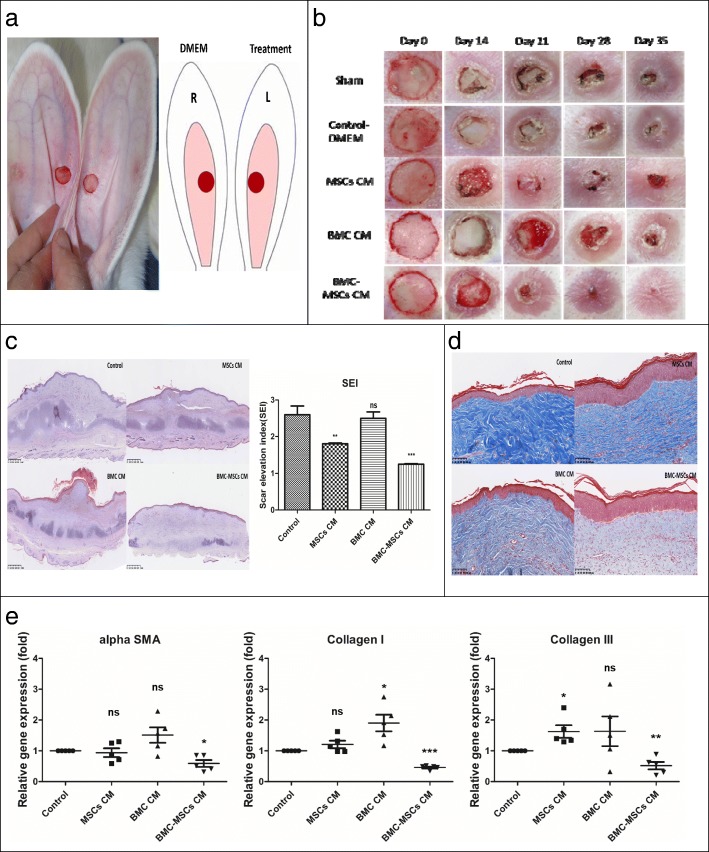
The rabbit ear HS model. BMC-induced MSC CM accelerated wound healing and prevented HS formation in our rabbit ear model. **a** Experimental design. Four groups of rabbits were used. MSC CM, BMC CM, or BMC-MSC CM was administered to the left ears. In one set of rabbits, the left ears were left untreated as a sham group. The right ears of all groups received a DMEM injection as an internal control. **b** Images of gross examination. **c** Histological analysis of wounds and scar elevation index (SEI) measurement. Cells were stained with hematoxylin and eosin. Original magnification × 40. The SEI was measured using cross-sections of the central area of the wound. **d** Masson trichrome staining was used for the evaluation of collagen fiber organization. **e** Effects of MSC CM, BMC CM, and BMC-induced MSC CM on *α-SMA*, collagen type Ι, and collagen III mRNA expression, as measured by quantitative polymerase chain reaction using *GADPH* as a housekeeping gene and internal standard control. The data are shown as means ± standard errors of the means. *P < 0.05; **P < 0.01; ***P < 0.001. MSC CM, mesenchymal stem cell-conditioned medium; BMC CM, bone marrow concentrate; DMEM, Dulbecco’s modified Eagle’s medium; L, left; R, right

The rabbit ear HS model was established as described previously [15]. Briefly, 12 adult female New Zealand albino rabbits (each weighing 3–4 kg) were given anesthesia under sterile conditions in preparation for wounding based on a protocol approved by the Animal Care and Experiment Committee of Chang Gung Memorial Hospital. Circular, full-thickness, 1-cm wounds were made to the bare cartilage on the ventral surface of each ear, and the epidermis, dermis, and perichondrium were carefully removed (Fig. 1a). The wounds were covered with neomycin ointment and cleaned of secretions the next day. Two weeks after the operation, the margin of the wound showed obvious protrusions as an indicator of HS formation. Two hundred microliters of DMEM, MSC CM, BMC CM, or BMC-induced MSC CM was injected into each lesion on days 14, 21, and 28 after the operation. We excluded samples with infected or necrotic wounds.

### Evaluation of scars

Photographs were acquired every week to observe external changes in wounds after the operation. The re-epithelization rate was recorded and analyzed using Image J software (National Institutes of Health, Bethesda, MD, USA).

### Histology and immunohistochemistry

Scars were harvested for histological detection 35 days after the operation, bisected, and immediately fixed in 10% formalin. The scars were embedded in paraffin and cut into sections. For analysis of the scar evaluation index (SEI), sections were stained with hematoxylin and eosin, examined under a microscope (Nikon Eclipse E400; Nikon, Tokyo, Japan), and evaluated with a digital image analysis system (Image J). Measurements were performed twice by a blinded examiner, and average values were used. Further evaluation of the collagen fiber arrangement was carried out using Masson trichrome staining. Briefly, the sections were fixed in Bouin’s solution for 1 h at 56 °C to improve staining quality and then rinsed in running tap water for 5–10 min to remove yellow color. Samples were then stained with Weigert’s iron hematoxylin working solution for 10 min, rinsed in running warm tap water for 10 min and Biebrich scarlet-acid fuchsin solution for 10–15 min, and placed in 1% phosphomolybdic-phosphotungstic acid solution for 10–15 min. The sections were then stained in aniline blue solution for 5 min and rinsed in 1% acetic acid solution for 2 min. Collagen fibers were stained blue. Keratin and muscle fibers were stained red. The cell cytoplasm and nuclei were stained light pink and dark brown, respectively. Regions were randomly selected and photographed.

### Treatment of scar-derived fibroblasts with MSCs, BMC, and BMC-induced MSC CM

Adherent scar-derived fibroblasts were incubated in serum-free medium containing transforming growth factor TGF β1 (10 ng/mL; Sigma-Aldrich; cat. no. H8541) for 24 h then switched the culture medium to DMEM, MSC CM, BMC CM, or BMC-induced MSC CM for 72 h. Cells were then subjected to comparative analyses.

### Cellular proliferation

A Cell Counting Kit-8 (CCK-8; Sigma-Aldrich) was used to determine the extent of fibroblast proliferation. Fibroblasts were seeded in 96-well plates at an initial concentration of 5000 cells/well. After stimulated with TGF-β1 (10 ng/mL) for 24 h, then shift the culture medium to DMEM, MSC CM, BMC CM, or BMC-induced MSC CM. CCK-8 reagent was added for 2 h after incubation at 37 °C for 0, 24, 48, or 72 h. The absorption rate and reference wavelength were measured at 450 nm.

### Collagen gel contraction assay

After stimulated with TGF-β1 for 24 h, scar-derived fibroblasts were seeded in 24-well plates at a density of 2 × 10^5^ cells/mL in 0.6 mL DMEM, MSC CM, BMC CM, or BMC-induced MSC CM supplemented with antibiotics, and 1 mg/mL acid-extracted collagen I from rat tails (Sigma-Aldrich) was added to different wells. The cells were cultured at 37 °C for 20 min to allow collagen polymerization. Then, 0.6 mL of the different treatment media was added to each group. After incubation at 37 °C for 60 min, the gels were released from the plates by tilting the plates. Contraction of collagen gels was monitored by measuring the gel area at 3, 6, 9, 12, and 24 h. Data are presented means ± standard errors of the means (SEMs) of three independent experiments, conducted in triplicate.

### Immunofluorescence analysis

Scar-derived fibroblasts were seeded at a density of 10,000 cells/cm^2^. The cells were then incubated in serum-free medium containing transforming growth factor TGF β1(10 ng/mL) for 24 h then shift the culture medium to DMEM, MSC CM, BMC CM, or BMC-induced MSC CM. For α smooth muscle actin (α-SMA) staining, cells were incubated with primary antibodies against α-SMA (1 μg/mL; Invitrogen) for 75 min at 25 °C, followed by incubation with a fluorescein isothiocyanate-488-conjugated secondary antibody. Cells were then stained with 4,6-diamidino-2-phenylindole (DAPI; 1 μg/mL; Sigma-Aldrich) for nuclear staining. Immunofluorescence signals were captured using a confocal microscope (LSM 510; METALaser Scanning Microscope; Zeiss).

### Western blotting

Immunoblot procedures were performed as described previously [16]. As primary antibodies, mouse anti-α-SMA (2 μg/mL; Invitrogen), anti-GADPH (0.05 μg/mL; Sigma-Aldrich), rabbit anti-collagen I (1:1000; Abcam, Cambridge, UK; cat. no. ab34710), rabbit anti-collagen III (1:5000; Abcam; cat. no. ab7778), rabbit anti-fibronectin (1:1000; Abcam; cat. no. 1b45688), anti-matrix metalloproteinase (MMP)-1 (1:1000; Abcam; cat. no. ab134184), and anti-MMP-13 (1:1000; Abcam; cat. no. ab51072) were used. Anti-mouse IgG (1:50000; Sigma-Aldrich) and anti-rabbit IgG (1:10000 Sigma-Aldrich) were used as secondary antibodies. Photographs were acquired with an Amersham Imager 600 (GE Healthcare). Densitometric analysis was performed using Image Quant TL software. Expression of proteins of interest was normalized to the expression of GADPH.

### RNA isolation, quantitative real-time reverse transcription polymerase chain reaction (qRT-PCR), and preparation for RNA-seq

qRT-PCR was performed as previously reported [16]. Briefly, scar-derived fibroblasts were collected after 72 h of incubation with different treatments, and rabbit scars were harvested on day 35 post-operation. Total RNA was extracted using an RNA isolation kit (Qiagen, Hilden, Germany), and RNA purity was evaluated by calculating the A260/A280 ratio by nanodrop spectrophotometer, aiming for a value of 1.8–2.1. The primer pairs used for gene amplification from the cDNA template are listed in Additional file 2. The results from three independent reactions were used to determine the relative expression levels of the target genes, which were normalized to the expression level of glyceraldehyde 3-phosphate dehydrogenase (GAPDH) as a control. As for RNA sequencing, sample quality control of RNA integrity was performed using an Agilent 2100 Bioanalyzer—RNA 6000 Nano kit. High-quality RNA was selected based on samples with an RNA integrity number over 9.5. Qualified samples were then used to construct RNA-seq cDNA libraries following the standard Illumina protocol. cDNA libraries were quantified by qRT-PCR using a Roche LightCycler 480 system and Qubit Fluorometer (Invitrogen). The cDNA libraries were pooled and sequenced on an Illumina NextSeq 500 platform with single-end 76-bp reads.

### RNA-seq analysis

Quality control of RNA-seq reads was performed using FASTX-Toolkit (v0.0.13.2) with high base calling quality (Phred quality score over 20) and RNA read lengths over 35 bases. Reads were mapped to the UCSC human genome hg38 using Tophat2 (v2.1.0) [17]. The transcripts and abundances were assembled and estimated by Cufflinks (v2.2.1) [18]. Mapping reads of each gene were counted by HTSeq (v0.11.1) [19]. Differential gene expression was analyzed using DESeq2 (v1.22.2) [20]. Genes with low baseMean values (≤ 10) were filtered. Differences with *P* values of less than 0.05 were considered statistically significant. Genes were identified as upregulated or downregulated if the fold change between case and control sample was greater than |1.3|.

### Gene ontology (GO) and enrichment analysis

To study the functions of differentially expressed genes (DEGs), functional groups and pathways encompassing the DEGs were identified based on GO and Kyoto Encyclopedia of Genes and Genomes Pathway analysis using the Database for Annotation, Visualization, and Integrated Discovery (v.6.8) software [21, 22]. The threshold was set as modified Fisher exact *P* value (EASE score) ≤ 0.1, and the top 20 functional terms sorted by modified Fisher exact *P* values are shown.

### Statistical analysis

Statistical differences were calculated using two-tailed Student’s *t* tests compared to the control group. Differences with *P* values of less than 0.05 were considered statistically significant. For RNA sequencing and GO analysis, the statistical analysis was described as above.

## Results

### BMC-induced MSC CM significantly improved wound healing and reduced HS formation after wound healing in a rabbit ear model

The experimental groups and study design are shown in Fig. 1a. Rabbit ears were treated with DMEM, MSC CM, BMC CM, or BMC-induced MSC CM on days 14, 21, and 28 after the operation. Rabbit ears treated with BMC-induced MSC CM healed significantly faster than rabbit ears in the other groups, and complete closure occurred on day 28 after the operation. However, sham-, DMEM-, MSC CM-, and BMC CM-treated rabbit ears did not show complete re-epithelization within the time course tested (Fig. 1b). An additional file shows the time of rabbit ear wound healing in more detail (see Additional file 3). On postoperative day 35, hematoxylin-and-eosin staining of cross-sections of HSs showed that BMC-induced MSC CM- and MSC CM-treated scars were flatter and thinner. However, DMEM- and BMC CM-treated scars were obviously thickened, with slight contraction (Fig. 1c). The SEIs in MSC CM- and BMC-induced MSC CM-treated scars were much lower than those in internal controls. However, there were no significant differences between DMEM- and BMC CM-injected scars (Fig. 1c).

### BMC-induced MSC CM attenuated scar-forming profibrotic activity and fibroblast activation in a rabbit ear model

Profibrotic events and fibroblast activation are key processes in HS formation and can be measured by evaluating collagen deposition and myofibroblast numbers. Masson trichrome staining revealed that collagen fibers were dense and irregularly arranged in DMEM- and BMC CM-treated scars. Conversely, collagen deposition was reduced and well arranged in MSC CM- and BMC-induced MSC CM-treated scars (Fig. 1d). Detection of myofibroblasts using α-SMA showed that *α-SMA* gene expression in scar tissue was reduced in BMC-induced MSC CM-treated scars compared with those in DMEM- and MSC CM-treated scars on day 35 post-operation (Fig. 1e). Collagen I and collagen III gene expression levels were also significantly reduced in BMC-induced MSC CM-treated scars compared with that in DMEM-treated scars (Fig. 1e).

### BMC-induced MSC CM promoted the balance of ECM turnover in human HS-derived fibroblasts

**Fig. 2:**
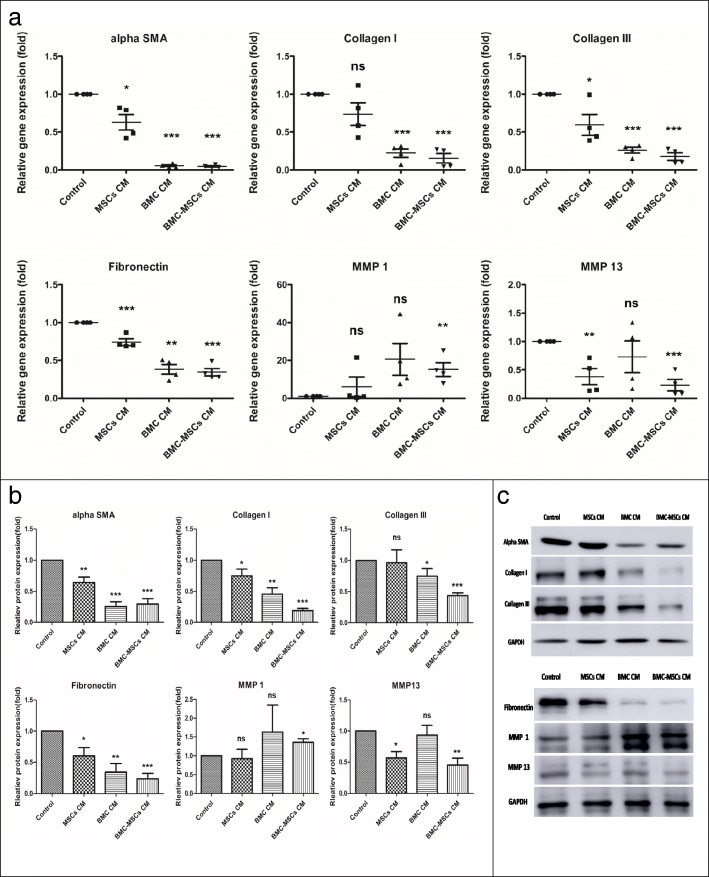
Effects of MSC CM, BMC CM, and BMC-induced MSC CM on cultured primary HSs. **a** qRT-PCR analysis of *α-SMA*, collagen I, collagen III, fibronectin, *MMP-1*, and *MMP-13* in HSs treated for 72 h with different stimuli. Expression is reported relative to that of *GAPDH*. Each data point was normalized to *GAPDH* expression and then the value of the control (DMEM), which was arbitrarily set as 1. **b**, **c** Western blot analysis and quantitative data for α-SMA, collagen I, collagen II, fibronectin, MMP-1, and MMP-13 after treatment with DMEM, MSC CM, BMC CM, and BMC-induced MSC CM for 72 h. GAPDH served as a control for equal protein loading. Histogram summarizing the results in **b**. Results were from three independent experiments using cells from four patients with HSs. The data are shown as means ± standard errors of the means. *P < 0.05; **P < 0.01; ***P < 0.001

During normal wound healing, matrix metalloproteinase (MMP) expression modulates matrix turnover [23]. MMPs catalyze the hydrolysis of ECM molecules [24], and MMP-13 and MMP-1 expression levels vary between normal and abnormal scar tissues [25]. Therefore, we examined the effects of BMC-induced MSC CM on ECM synthesis in active human HS-derived fibroblasts. BMC-induced MSC CM decreased collagen I, collagen III, and fibronectin expression at both the transcriptional and translational levels (Fig. 2). Moreover, BMC-induced MSC CM significantly increased MMP-1 expression and decreased MMP-13 expression.

To further determine the functional consequences of these effects, we analyzed GO data for BMC-induced MSC CM-treated HSs from three human samples. Genes regulating ECM formation, ECM organization, extracellular structure organization, collagen fibril organization, and collagen fibril binding, such as collagen type VIII alpha 2 (COL8A2), collagen type XI alpha 1 (COL11A1), collagen Type XIV Alpha 1 (COL14A1), collagen Type XVI alpha 1 (COL16A1), extracellular matrix protein 2 (ECM2), matrilin 3 (MATN3), and matrix metalloproteinase 13 (MMP-13), as well as related pathways were downregulated (Fig. 5d, f). Additionally, *MMP-1* was upregulated (Fig. 5e). An additional file shows RNA sequence data in more detail (see Additional file 4). Taken together, our results showed that BMC-induced MSC CM reversed the abnormal expression profiles of collagens and MMPs in human HS-derived fibroblasts.

### BMC-induced MSC CM reduced the contractile ability of HS in collagen gels

**Fig. 3:**
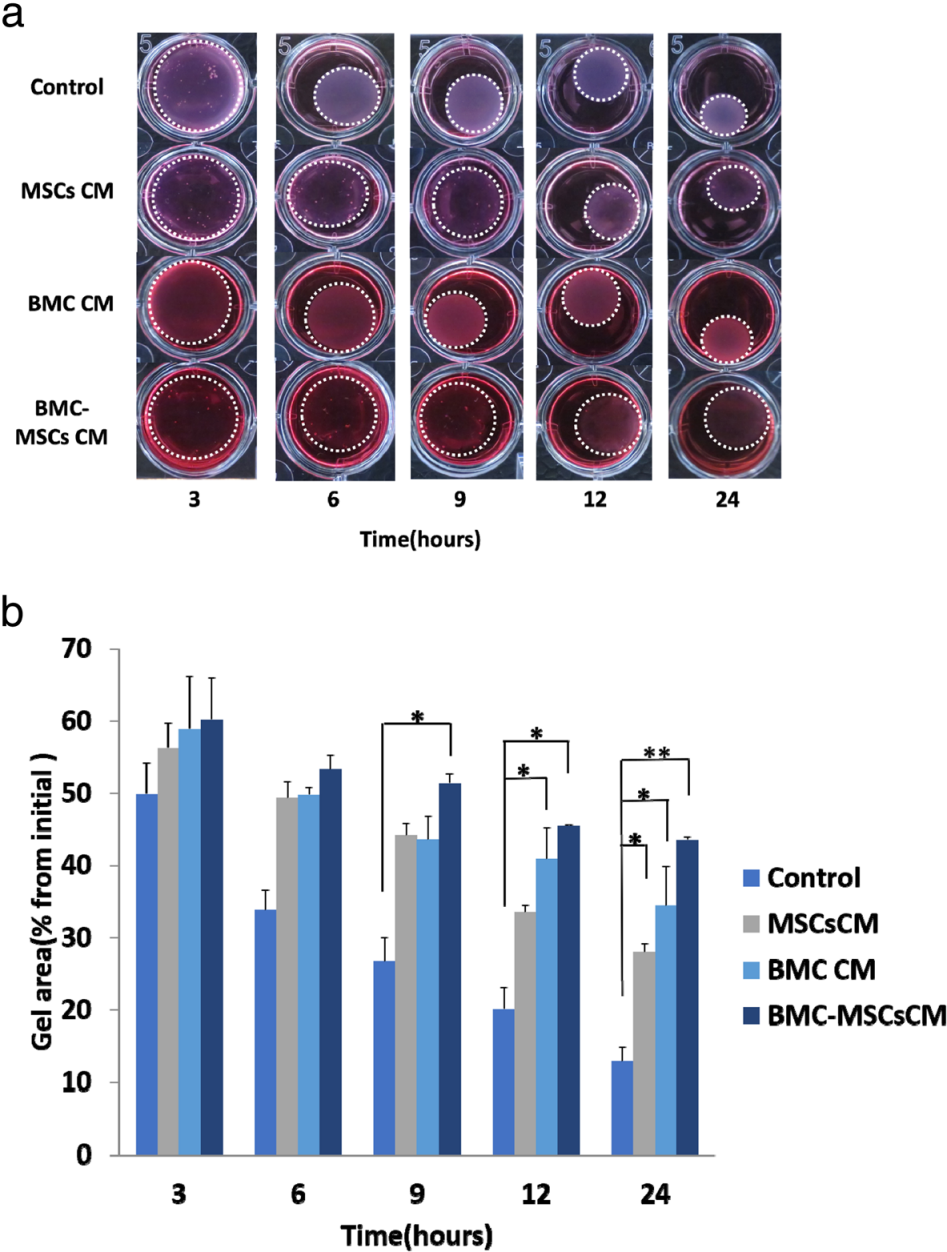
Effects of MSC CM, BMC CM, and BMC-induced MSC CM attenuated the contractile ability of human hypertrophic scar-derived fibroblasts in 3D collagen gels. **a**, **b** Representative images and quantification of collagen gel contraction after treatment with different samples for 3, 6, 9, 12, or 24 h. The white spots outline the gels (*n* = 3). Values are shown as means ± standard errors of the means. *P < 0.05, **P < 0.01

To investigate the effects of BMC-induced MSC CM on the contractile ability and functions of HSs, we next examined the properties of human HS-derived fibroblasts in three-dimensional (3D) fibroblast function assays. After edges were liberated from array wells, we observed collagen lattice contraction at 3, 6, 9, 12, and 24 h. BMC-induced MSC CM had significant time-dependent inhibitory effects on the contractile ability of HSs (Fig. 3a, b). No differences in collagen contraction were observed at 3 and 6 h between any of the groups. At 9, 12, and 24 h, BMC-induced MSC CM induced the lowest collagen contraction, followed by BMC CM, MSC CM, and DMEM. BMC-induced MSC CM and BMC CM resulted in significantly lower collagen matrix contraction compared with that in the control group at 12 and 24 h. Thus, MSC CM, BMC CM, and BMC-induced MSC CM efficiently inhibited the contractile ability of HSs in 3D collagen gels, and BMC-induced MSC CM was more effective than the other treatments.

### BMC-induced MSC CM reversed the transformation of fibroblasts to myofibroblasts in human HS-derived fibroblasts treated with TGF-β1

**Fig. 4:**
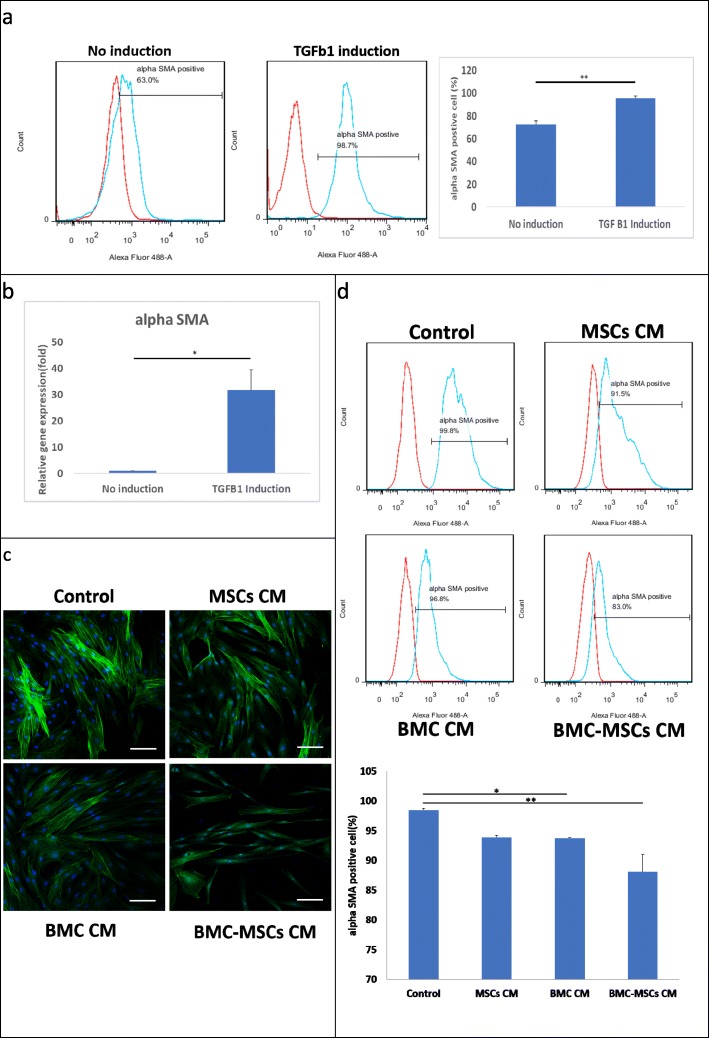
BMC-induced MSC CM attenuated the activation of TGF-β1-stimulated myofibroblasts in human hypertrophic scar-derived fibroblasts. **a** Flow cytometry analysis and quantitative data for α-SMA-positive cells after TGF-β1 stimulation for 24 h. **b** Expression of the *α-SMA* gene after TGF-β1 stimulation for 24 h. **c** Immunofluorescence staining for α-SMA expression in HSs stimulated with TGF-β1 for 24 h and treated with DMEM, MSC CM, BMC CM, and BMC-induced MSC CM for 72 h. Scale bars = 100 μm. DAPI, 4,6-diamidino-2-phenylindole; MSC CM, mesenchymal stem cell-conditioned medium; BMC CM, bone marrow concentrate; DMEM, Dulbecco’s modified Eagle’s medium; SMA, smooth muscle actin. **d** Flow cytometry analysis and quantitative data for α-SMA-positive cells after TGF-β1 stimulation for 24 h, followed by treatment with DMEM, MSC CM, BMC CM, and BMC-induced MSC CM for 72 h (*n* = 4). Values are denoted as means ± standard errors of the means. *P < 0.05, **P < 0.01

In wound healing, TGF-β1 induces the differentiation of fibroblasts into myofibroblasts, which contract the wound and facilitate remodeling of the ECM [24, 26, 27]. To investigate the phenotype of fibroblasts isolated from human HSs, we examined α-SMA expression in these cells by flow cytometry. Only 60–80% of fibroblasts from HSs were positive for α-SMA. After stimulation with TGF-β1 (10 ng/mL) for 24 h, more than 98% of fibroblasts from HSs were positive for α-SMA (Fig. 4a). *α-SMA* gene expression was also significantly increased compared with that in untreated cells (Fig. 4b). To further investigate the effects of BMC-induced MSC CM on fibroblast activation in vitro, we evaluated the levels of α-SMA expression in cultured HSs treated with 10 ng/mL TGF-β1 for 24 h followed by different treatments for 72 h. We found that α-SMA gene and protein expression were noticeably reduced in the BMC-induced MSC CM-, BMC CM-, and MSC CM-treated groups compared with that in the DMEM-treated group (Fig. 2). Immunostaining and flow cytometry analyses also showed that BMC-induced MSC CM suppressed TGF-β1-induced α-SMA expression and decreased α-SMA-positive fibroblasts by 10.4% ± 2.2% (*P* < 0.01) in cultured HSs (Fig. 4c, d). These findings suggested that BMC-induced MSC CM could reverse the activation of fibroblasts induced by the inflammatory factor TGF-β1.

### BMC-induced MSC CM accelerated cell proliferation in human HS-derived fibroblasts and our rabbit ear model

**Fig. 5:**
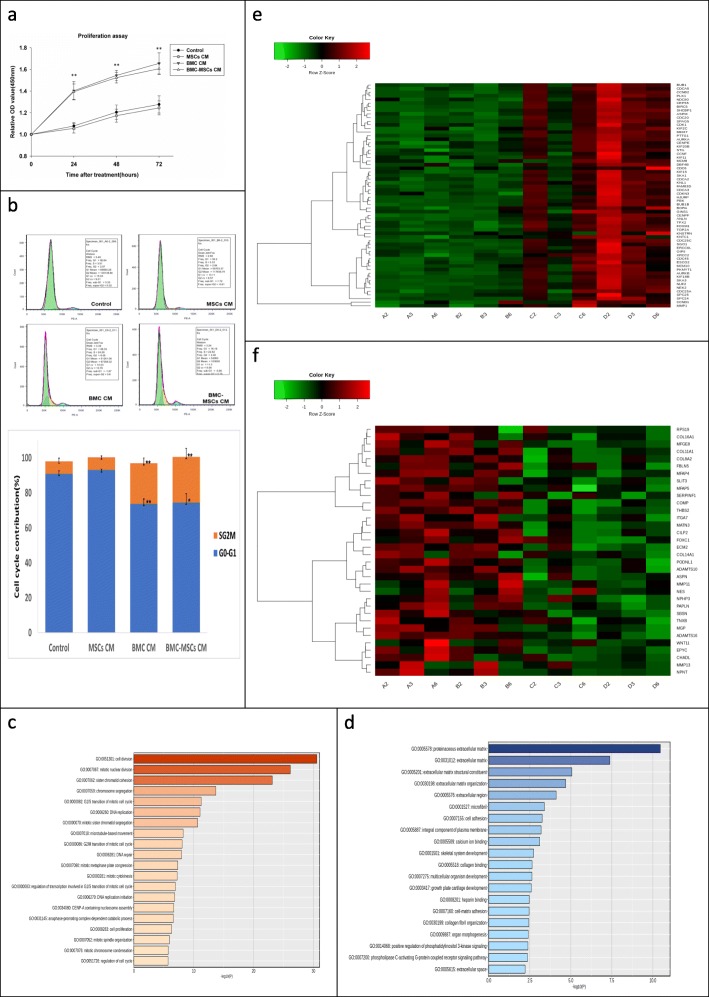
BMC-induced MSC CM accelerated cell proliferation in cell culture and in our rabbit ear model. **a** HS-derived fibroblasts were cultured in MSC CM, BMC CM, and BMC-induced MSC CM for 72 h. Cell viability was determined by CCK-8 assays every 24 h and compared with DMEM-treated samples. **b** Determination of cell cycle distribution by flow cytometry in HS-derived fibroblasts after culture with DMEM, MSC CM, BMC CM, or BMC-induced MSC CM for 72 h. **c**–**f** RNA-seq analysis of HSs treated with DMEM or BMC-induced MSC CM for 72 h. Genes that were differentially expressed between DMEM and BMC-induced MSC CM are shown as up- or downregulated. **c**, **d** GO enrichment for up- and downregulated pathways. **e**, **f** Heatmap of hierarchical clustering indicated differentially expressed genes (rows) from three patients (fold change > 1.3, *P* < 0.05). Red indicates upregulation, and green indicates downregulation. Data are means ± standard errors of the means. *P < 0.05 compared with DMEM. A, DMEM; B, MSC CM; C, BMC CM; D, BMC-MSC CM; 2, 3, 6, no. of patient

CCK-8 assays showed that the proliferation of human HS-derived fibroblasts increased after incubation with BMC CM or BMC-induced MSC CM for 24, 48, or 72 h, compared with that in cells incubated with DMEM or MSC CM (Fig. 5a). Additionally, flow cytometry showed that in the BMC CM and BMC-induced MSC CM groups, the number of cells in the G_0_/G_1_ phase was significantly decreased, whereas the numbers of cells in the S and G_2_/M phases were significantly increased (Fig. 5b). Furthermore, GO analysis revealed genes related to cell proliferation and DNA replication, such as SCL-interrupting locus protein (STIL), cell division cycle (CDC), kinesin family member (KIF), marker of proliferation Ki-67, and DNA replication complex GINS protein, were upregulated in BMC-induced MSC CM-treated HSs. Pathways related to cell division and mitosis were upregulated in this cohort as well (Fig. 5c, e). Interestingly, in rabbit ear HSs, the BMC-induced MSC CM-treated group showed high levels of DNA oxidation compared with those in the DMEM-, MSC CM-, and BMC CM-treated groups, as measured by 8-oxodG assays (Additional file 5), suggesting that the resident cells in rabbit ear wounds underwent DNA replication and proliferation.

## Discussion

Several factors contribute to HS formation during the wound healing process; for example, prolonged wound healing, trauma mechanism and severity, and mechanical tension on the wound are leading causes of HS formation [28]. Importantly, the timing of wound re-epithelization can decide the outcome of the healing process [29]. Slow epithelization of tissues over the wounded area results in HS formation, whereas rapid epithelization results in regeneration [30, 31]. Delayed wound healing increases the chance of HS formation. Our results showed that BMC-induced MSC CM accelerated re-epithelization and resulted in much lower SEI, more regular collagen arrangement, and decreased α-SMA, collagen type Ι, and collagen type III expression in our rabbit ear model. Moreover, cell proliferation was elevated in the BMC-induced MSC CM group.

The wound healing process consists of three phases: inflammation, proliferation, and remodeling. During the initial wound inflammation stage, neutrophils secrete reactive oxygen species (ROS) to sterilize the wound. In tissues with prolonged inflammation, ROS are overproduced, and T cells and macrophages are dysfunctional [1, 32]. During the proliferation stage, TGF-β1 plays a critical role. TGF-β1 is a collagen-stimulating factor that can inhibit MMP expression and contribute to ECM deposition within wounds [33, 34]. TGF-β1 can also stimulate the transformation of fibroblasts into myofibroblasts at a later stage, which can narrow the margin of the wound by contraction and therefore accelerate re-epithelization. However, a prolonged proliferation stage will lead to excessive irregularly arranged collagen bundles in the ECM and cause tensed, over contracted scars as additional pathological characteristics of HSs [35, 36]. During the remodeling stage, type III collagen, which is prevalent during proliferation, is replaced by type I collagen. Additionally, disorganized collagen fibers are rearranged, crosslinked, and aligned along tension lines. All protein components and the ECM are subjected to degradation and modification. The most important enzymes in ECM remodeling are MMPs [37]. If protein components in the ECM fail to be degraded, HSs will develop. Our results showed that BMC-induced MSC CM could decrease α-SMA gene and protein expression and reverse myofibroblast activation stimulated by TGF-β1. BMC-induced MSC CM also maintained tissue homeostasis by regulating the turnover of the ECM. Gene and protein expression of MMP-1 was elevated, whereas that of MMP-13 was decreased in HSs after treatment with BMC-induced MSC CM. The expression of collagen types I and III was also decreased after treatment with BMC-induced MSC CM in cultured HS-derived fibroblasts and our rabbit ear model. Overall, our observations suggested that BMC-induced MSC CM may suppress HS formation by modulating the proliferation and remodeling stages, reducing profibrotic gene activation, reversing myofibroblast activation, and facilitating ECM turnover and degradation; these changes together attenuated HS formation.

MSCs have anti-scarring effects by promoting wound healing. Additionally, MSCs have strong therapeutic effects on HSs because these cells can interact with many types of cells in the microenvironment through paracrine signaling pathways [38]. Components secreted from MSCs, including molecules and extracellular vesicles, are responsible for these therapeutic effects and have both local and distant effects. However, this process is not spontaneous and can be altered by different stimuli [39]. Several studies have shown that MSC-based strategies should be combined with appropriate differentiation and growth factors to obtain the maximum therapeutic effects from MSCs [39–42]. Different preconditioning strategies can enhance the secretion of therapeutic factors into the MSC culture medium [42, 43]. Thus, it is necessary to determine which culture conditions allow MSCs to produce differing sets of trophic factors [39].

BMC is currently often used for regenerative medicine because it contains a heterogenous mixture of cells, including small amount MSCs, hematopoietic cells, and platelets, as well as bioactive molecules, such as platelet-derived growth factor, basic fibroblast growth factor, insulin-like growth factor-I, and vascular endothelial growth factor [3, 41, 44]. BMC acts as a storage vehicle for a variety of growth factors, thereby enhancing the differentiation of MSCs and stimulating the production of secreted factors from MSCs. We used BMC from rabbits to stimulate mouse MSCs, and the CM we obtained was shown to have significant effects on rabbit ear and human HS fibroblasts, despite the use of mixed species. MSCs are known to have immunomodulatory effects and cross-species immunotolerance [45]; however, the mechanisms mediating the differences between secreted factors from mouse MSCs with and without rabbit BMC stimulation are still unclear. Accordingly, in our future studies, we will investigate the differential contents of CM (BMC-induced MSC CM versus MSCs and/or BMC CM) and the contributions of rabbit BMC cells, mouse MSCs, and their derivatives in BMC-induced MSC CM.

## Conclusions

BMC-induced MSC CM accelerated wound healing and attenuated HS formation in a rabbit ear model. Further analyses indicated that BMC-induced MSC CM had anti-HS effects by increasing cell proliferation, modulating the proliferation and remodeling phases of the wound healing process, reducing profibrotic gene expression, balancing ECM turnover, and reversing myofibroblast activation. Therefore, BMC-induced MSC CM may be a promising candidate for preventing HS formation and treating fibrotic diseases.

## Additional files

Additional file 1: Mouse bone marrow MSC isolation. The femurs and tibias of Balb/c mice were dissected, and the ends of the bones were cut. Approximately 2 mL of bone marrow aspirate was collected into a syringe containing 4000 U heparin to prevent clotting. The bone marrow sample was washed with phosphate-buffered saline (PBS), and the cells were recovered after centrifugation at 900×g for 10 min. The cells were resuspended in 10 mL PBS followed by filtration through a cell strainer with 70-μm nylon mesh. To isolate the mononuclear cells, the filtered bone marrow cells were added to 10 mL Ficoll-Paque PLUS density gradient separation medium (density: 1.077 g/mL) and centrifuged at 18 °C for 30 min at 1100×g. The mononuclear cells were collected, washed with PBS, and centrifuged for 10 min at 900×g. The cells were resuspended, counted, plated at 200000 cells/cm^2^, and cultured in Dulbecco’s modified Eagle’s medium containing 10% fetal bovine serum. The medium was replaced every 3 days, and the nonadherent cells were discarded. Cells were harvested at 80–90% confluence and then expanded. After a series of passages, the attached marrow cells became homogeneous. The quality and purity of obtained MSCs were then assessed by detecting the expressions of CD29, CD44, CD73, CD105, CD106 and Sca1, using flow cytometry (FACScan flow cytometer; Becton Dickinson) [10, 12]. The isolated MSCs were able to be differentiated into bone, adipose tissue, and hepatocyte in vitro [10, 12]. (DOC 2510 kb)

Additional file 2: List of oligonucleotides used for quantitative Reverse-Transcriptase polymerase Chain Reaction. (DOC 36 kb)

Additional file 3: Time of rabbit ear wound healing. The affected skin area after the 1 cm^2^ wound creation. Rabbit ears treated with BMC-induced MSCs CM healed significantly faster than rabbit ears in the other groups, and complete closure occurred on day 28 after operation. However, sham-, DMEM-, MSCs CM-, and BMC CM-treated rabbit ears did not show complete re-epithelization within the time course tested. A two-tailed independent t-test was performed to evaluate the significant difference between groups. * *p* < 0.05. ** *p* < 0.01. Error bar indicates standard deviation. (DOC 173 kb)

Additional file 4: Raw data of RNA sequence. Detail of differential gene expression and fold of change between case and control. A: DMEM, B: MSCs CM, C: BMC CM, D: BMC-MSCs CM. 2,3,6: No. of patient (XLSX 40 kb)

Additional file 5: DNA oxidation level- 8′-oxodG. Measurement of 8′-oxodG in rabbit hypertrophic scars on day 35 after treatment with DMEM, MSCs CM, BMC CM, or BMC-induced MSCs CM. BMC-induced MSCs CM-treated group showed high levels of DNA oxidation compared with those in the DMEM-, MSC CM-, and BMC CM-treated groups. (* p < 0.05) Error bar indicates standard deviation. (DOC 120 kb)

## Data Availability

The datasets used and/or analyzed during the current study are available from the corresponding author on reasonable request.

## Abbreviations

α-SMA: α smooth muscle actin
BMC: Bone marrow concentrate
CCK-8: Cell counting kit-8
CM: Conditioned medium
HS: Hypertrophic scar
MSC: Mesenchymal stem cell
TGF β1: Transforming growth factor β1
