# Supplementary material for: Bone marrow concentrate-induced mesenchymal stem cell conditioned medium facilitates wound healing and prevents hypertrophic scar formation in a rabbit ear model
Source: Stem Cell Res Ther. 2019 Aug 28;10:275. doi: 10.1186/s13287-019-1383-x (PMC6714083; doi:10.1186/s13287-019-1383-x)
Supplement: Supplementary file 1 — Mouse bone marrow MSC isolation. The femurs and tibias of Balb/c mice were dissected, and the ends of the bones were cut. Approximately 2 mL of bone marrow aspirate was collected into a syringe containing 4000 U heparin to prevent clotting. The bone marrow sample was washed with phosphate-buffered saline (PBS), and the cells were recovered after centrifugation at 900×g for 10 min. The cells were resuspended in 10 mL PBS followed by filtration through a cell strainer with 70-μm nylon mesh. To isolate the mononuclear cells, the filtered bone marrow cells were added to 10 mL Ficoll-Paque PLUS density gradient separation medium (density: 1.077 g/mL) and centrifuged at 18 °C for 30 min at 1100×g. The mononuclear cells were collected, washed with PBS, and centrifuged for 10 min at 900×g. The cells were resuspended, counted, plated at 200000 cells/cm2, and cultured in Dulbecco’s modified Eagle’s medium containing 10% fetal bovine serum. The medium was replaced every 3 days, and the nonadherent cells were discarded. Cells were harvested at 80–90% confluence and then expanded. After a series of passages, the attached marrow cells became homogeneous. The quality and purity of obtained MSCs were then assessed by detecting the expressions of CD29, CD44, CD73, CD105, CD106 and Sca1, using flow cytometry (FACScan flow cytometer; Becton Dickinson) [10, 12]. The isolated MSCs were able to be differentiated into bone, adipose tissue, and hepatocyte in vitro [10, 12]. (DOC 2510 kb) [file 13287_2019_1383_MOESM1_ESM.doc]

**Additional file 1. Mouse bone marrow MSC isolation**

The femurs and tibias of Balb/c mice were dissected, and the ends of the bones were cut. Approximately 2 mL of bone marrow aspirate was collected into a syringe containing 4,000 U heparin to prevent clotting. The bone marrow sample was washed with phosphate-buffered saline (PBS), and the cells were recovered after centrifugation at 900 × *g* for 10 min. The cells were resuspended in 10 mL PBS followed by filtration through a cell strainer with 70-μm nylon mesh. To isolate the mononuclear cells, the filtered bone marrow cells were added to 10 mL Ficoll-Paque PLUS density gradient separation medium (density: 1.077 g/mL) and centrifuged at 18°C for 30 min at 1100 × *g*. The mononuclear cells were collected, washed with PBS, and centrifuged for 10 min at 900 × *g*. The cells were resuspended, counted, plated at 200000 cells/cm^2^, and cultured in Dulbecco’s modified Eagle’s medium containing 10% fetal bovine serum. The medium was replaced every 3 days, and the nonadherent cells were discarded. Cells were harvested at 80–90% confluence and then expanded. After a series of passages, the attached marrow cells became homogeneous. The quality and purity of obtained MSCs were then assessed by detecting the expressions of CD29, CD44, CD73, CD105, CD106 and Sca1, using flow cytometry (FACScan flow cytometer; Becton Dickinson)(10-13). The isolated MSCs were able to be differentiated into bone, adipose tissue, and hepatocyte in vitro (10-13).

| a  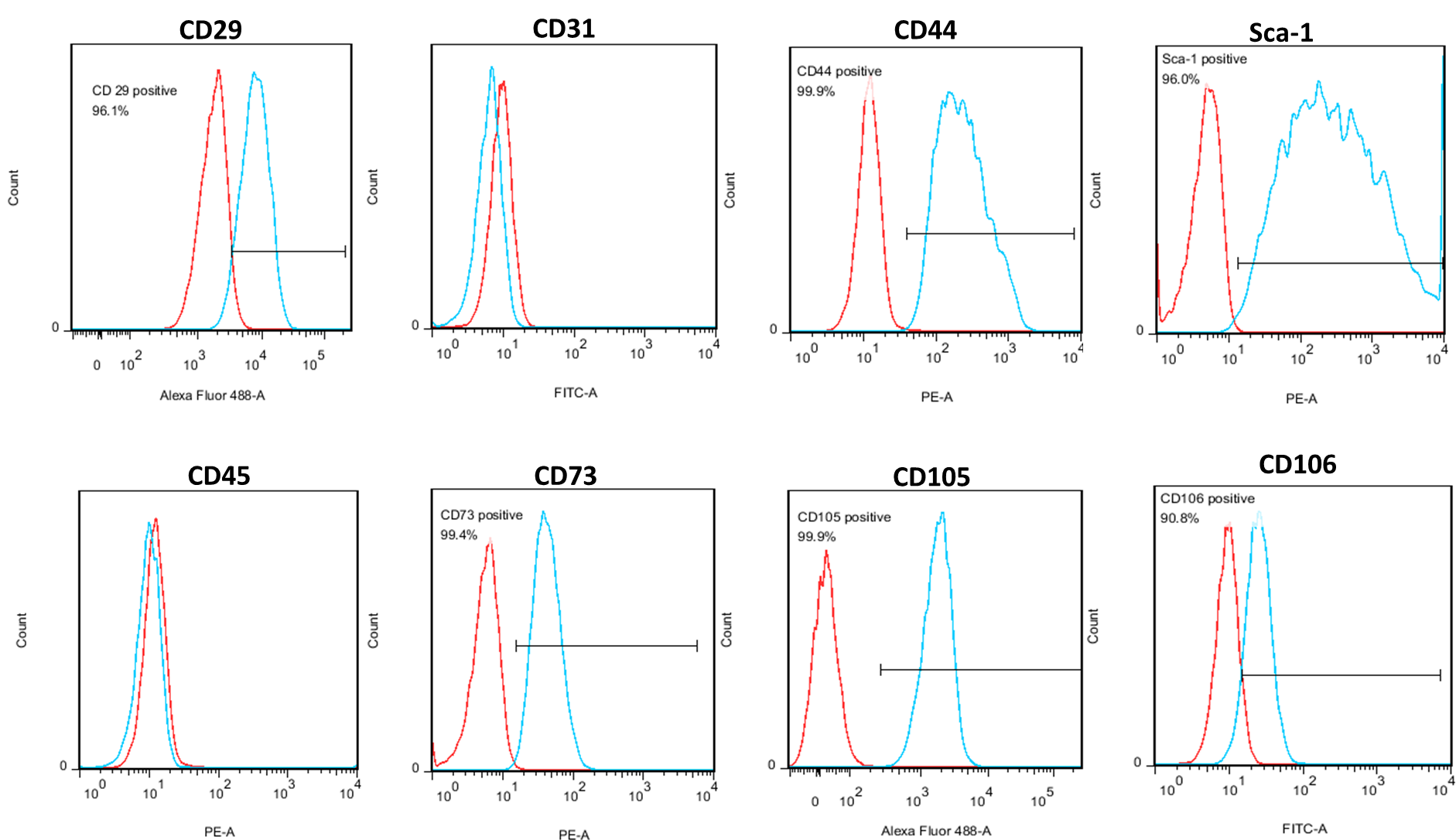 |
| --- |
| b  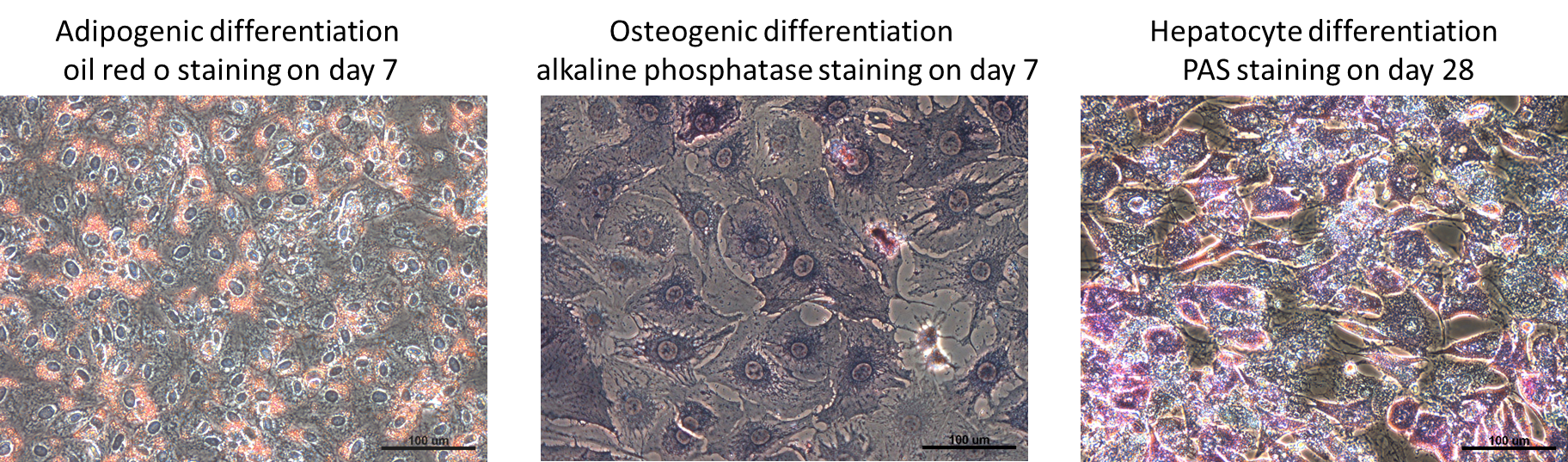 |
