# Supplementary material for: Bone marrow concentrate-induced mesenchymal stem cell conditioned medium facilitates wound healing and prevents hypertrophic scar formation in a rabbit ear model
Source: Stem Cell Res Ther. 2019 Aug 28;10:275. doi: 10.1186/s13287-019-1383-x (PMC6714083; doi:10.1186/s13287-019-1383-x)
Supplement: Supplementary file 2 — List of oligonucleotides used for quantitative Reverse-Transcriptase polymerase Chain Reaction. (DOC 36 kb) [file 13287_2019_1383_MOESM2_ESM.doc]

| Human | | |
| --- | --- | --- |
| Target gene | Sequence | Reference |
| Alpha SMA | F: 5’-ctgttccagccatccttcat-3’  R: 5’-tcatgatgctgttgtaggtggt-3’ | NM_001141945.2 |
| Collagen I | F: 5’-cccctggaaagaatggagat-3’  R: 5’-aatcctcgagcaccctgag-3’ | NM_000088.3 |
| Collagen III | F: 5’-ctggaccccagggtcttc-3’  R: 5’-catctgatccagggtttcca-3’ | NM_000090.3 |
| Fibronectin | F: 5’- ggctcgtgtgacagatgcta-3’  R:5’- cgtctcagtcttggttctcca-3’ | NM_212482 |
| MMP1 | F:5’- gctaacctttgatgctataactacga-3’  R:5’- tttgtgcgcatgtagaatctg-3’ | NM_002421 |
| MMP13 | F:5’- ccagtctccgaggagaaaca-3’  R:5’- aaaaacagctccgcatcaac-3’ | NM_002427 |
| GAPDH | F: 5’-ccatgttcgtcatgggtgt-3’  R: 5’-ccaggggtgctaagcagtt-3’ | NM_002046.6 |
| Rabbit | | |
| Target gene | Sequence | Reference |
| Alpha SMA | F: 5’-atcaccatcgggaacgaac-3’  R: 5’-gatgaaggagggctggaac-3’ | NM_001101682.2 |
| Collagen I | F: 5’-caacagaccggcaacctc-3’  R: 5’-gcagctcgatctcgttgg-3’ | XM_017348831.1 |
| Collagen III | F: 5’-cagggtgcagttggtagtcc-3’  R: 5’-gccactaggtccaacaggtc-3’ | XM_002712333.3 |
| GAPDH | F: 5’-cacagtttccatcccagacc-3’  R: 5’-tggtttcatgacaaggtaggg-3’ | NM_001082253.1 |

Additional file 2 List of oligonucleotides used for quantitative Reverse-Transcriptase polymerase Chain Reaction
