# Supplementary material for: Bone marrow concentrate-induced mesenchymal stem cell conditioned medium facilitates wound healing and prevents hypertrophic scar formation in a rabbit ear model
Source: Stem Cell Res Ther. 2019 Aug 28;10:275. doi: 10.1186/s13287-019-1383-x (PMC6714083; doi:10.1186/s13287-019-1383-x)

**Additional file 3.** **Time of rabbit ear wound healing**

The affected skin area after the 1 cm^2^ wound creation. Rabbit ears treated with BMC-induced MSCs CM healed significantly faster than rabbit ears in the other groups, and complete closure occurred on day 28 after operation. However, sham-, DMEM-, MSCs CM-, and BMC CM-treated rabbit ears did not show complete re-epithelization within the time course tested. A two-tailed independent t-test was performed to evaluate the significant difference between groups. * p < 0.05. ** p < 0.01. Error bar indicates standard deviation.


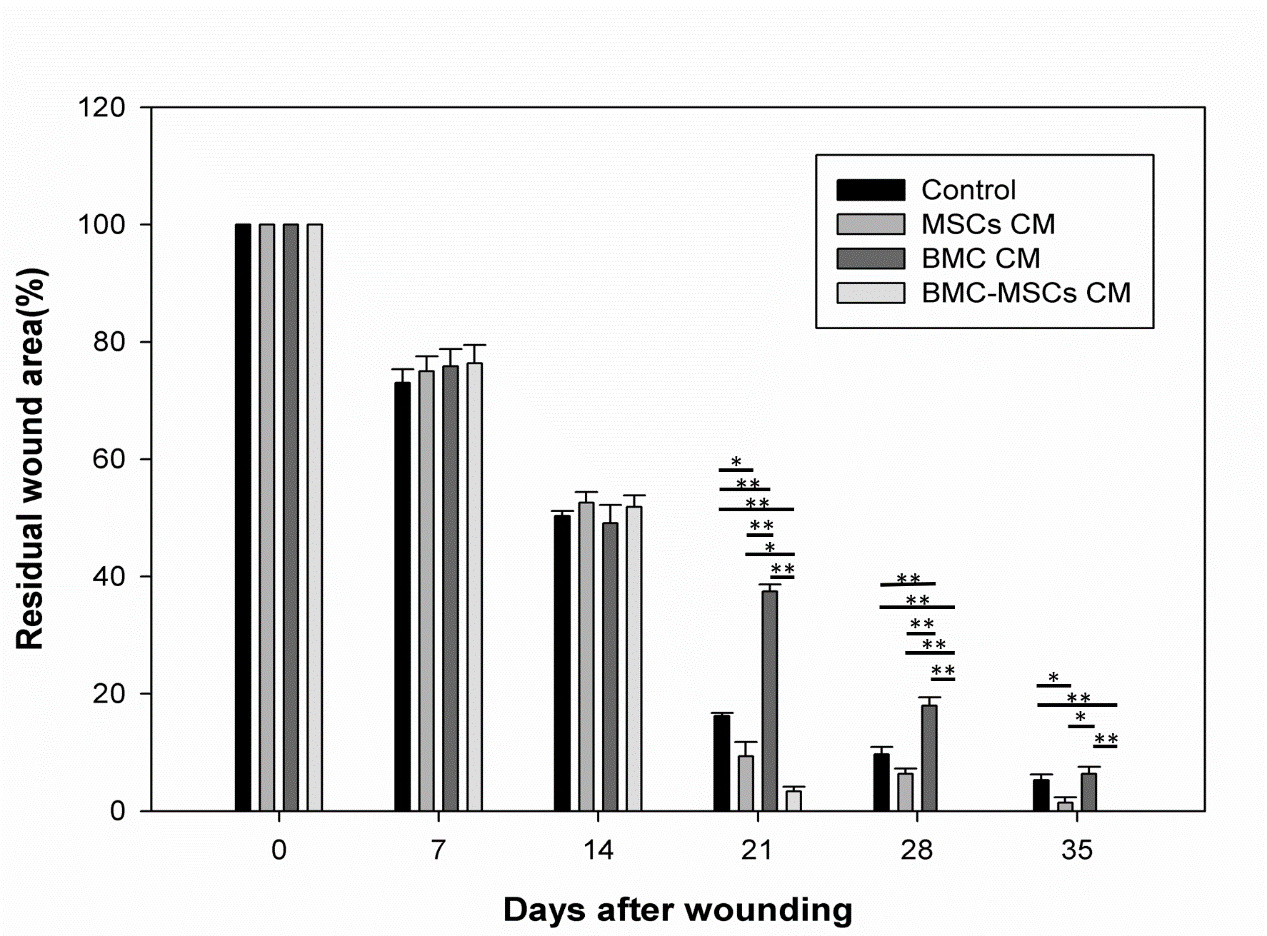

Supplement: Supplementary file 3 — Time of rabbit ear wound healing. The affected skin area after the 1 cm2 wound creation. Rabbit ears treated with BMC-induced MSCs CM healed significantly faster than rabbit ears in the other groups, and complete closure occurred on day 28 after operation. However, sham-, DMEM-, MSCs CM-, and BMC CM-treated rabbit ears did not show complete re-epithelization within the time course tested. A two-tailed independent t-test was performed to evaluate the significant difference between groups. * p < 0.05. ** p < 0.01. Error bar indicates standard deviation. (DOC 173 kb) [file 13287_2019_1383_MOESM3_ESM.doc]
