# Supplementary material for: Bone marrow concentrate-induced mesenchymal stem cell conditioned medium facilitates wound healing and prevents hypertrophic scar formation in a rabbit ear model
Source: Stem Cell Res Ther. 2019 Aug 28;10:275. doi: 10.1186/s13287-019-1383-x (PMC6714083; doi:10.1186/s13287-019-1383-x)

**Additional file 5. DNA oxidation level- 8’-oxodG**

Measurement of 8’-oxodG in rabbit hypertrophic scars on day 35 after treatment with DMEM, MSCs CM, BMC CM, or BMC-induced MSCs CM. BMC-induced MSCs CM-treated group showed high levels of DNA oxidation compared with those in the DMEM-, MSC CM-, and BMC CM-treated groups. (* p < 0.05) Error bar indicates standard deviation.


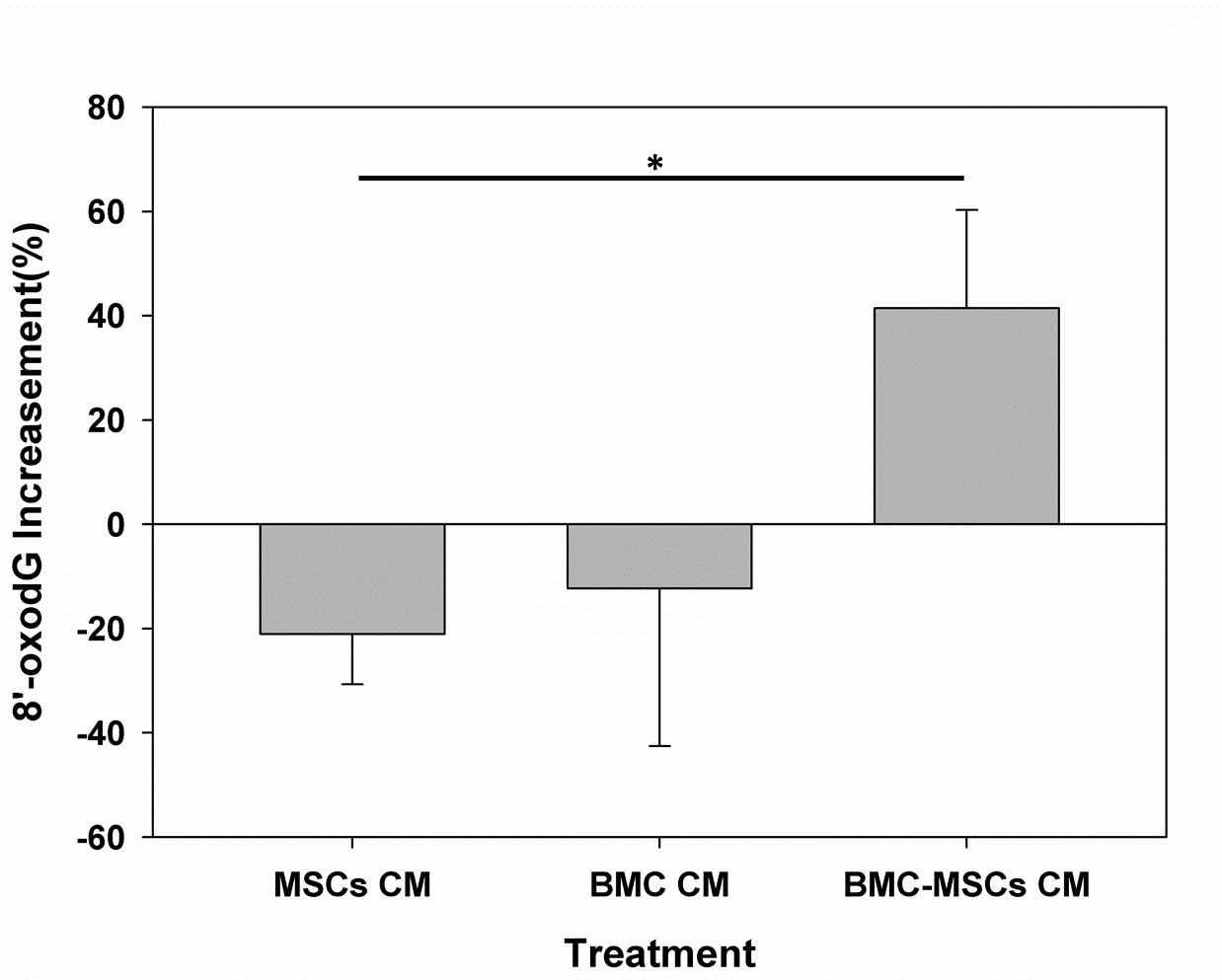

Supplement: Supplementary file 5 — DNA oxidation level- 8′-oxodG. Measurement of 8′-oxodG in rabbit hypertrophic scars on day 35 after treatment with DMEM, MSCs CM, BMC CM, or BMC-induced MSCs CM. BMC-induced MSCs CM-treated group showed high levels of DNA oxidation compared with those in the DMEM-, MSC CM-, and BMC CM-treated groups. (* p < 0.05) Error bar indicates standard deviation. (DOC 120 kb) [file 13287_2019_1383_MOESM5_ESM.doc]
